# Collaborative Human–Computer Vision Operative Video Analysis Algorithm for Analyzing Surgical Fluency and Surgical Interruptions in Endonasal Endoscopic Pituitary Surgery: Cohort Study

**DOI:** 10.2196/56127

**Published:** 2024-07-04

**Authors:** Chia-En Wong, Pei-Wen Chen, Heng-Jui Hsu, Shao-Yang Cheng, Chen-Che Fan, Yen-Chang Chen, Yi-Pei Chiu, Jung-Shun Lee, Sheng-Fu Liang

**Affiliations:** 1 Division of Neurosurgery Department of Surgery National Cheng Kung University Hospital Tainan Taiwan; 2 Department of Computer Science and Information Engineering National Cheng Kung University Tainan Taiwan; 3 Department of Cell Biology and Anatomy College of Medicine National Cheng Kung University Tainan Taiwan; 4 Department of Otolaryngology-Head and Neck Surgery National Cheng Kung University Hospital Tainan Taiwan; 5 Institute of Basic Medical Sciences College of Medicine National Cheng Kung University Tainan Taiwan; 6 Institute of Medical Informatics National Cheng Kung University Tainan Taiwan

**Keywords:** algorithm, computer vision, endonasal endoscopic approach, pituitary, transsphenoidal surgery

## Abstract

**Background:**

The endonasal endoscopic approach (EEA) is effective for pituitary adenoma resection. However, manual review of operative videos is time-consuming. The application of a computer vision (CV) algorithm could potentially reduce the time required for operative video review and facilitate the training of surgeons to overcome the learning curve of EEA.

**Objective:**

This study aimed to evaluate the performance of a CV-based video analysis system, based on OpenCV algorithm, to detect surgical interruptions and analyze surgical fluency in EEA. The accuracy of the CV-based video analysis was investigated, and the time required for operative video review using CV-based analysis was compared to that of manual review.

**Methods:**

The dominant color of each frame in the EEA video was determined using OpenCV. We developed an algorithm to identify events of surgical interruption if the alterations in the dominant color pixels reached certain thresholds. The thresholds were determined by training the current algorithm using EEA videos. The accuracy of the CV analysis was determined by manual review, and the time spent was reported.

**Results:**

A total of 46 EEA operative videos were analyzed, with 93.6%, 95.1%, and 93.3% accuracies in the training, test 1, and test 2 data sets, respectively. Compared with manual review, CV-based analysis reduced the time required for operative video review by 86% (manual review: 166.8 and CV analysis: 22.6 minutes; *P*<.001). The application of a human-computer collaborative strategy increased the overall accuracy to 98.5%, with a 74% reduction in the review time (manual review: 166.8 and human-CV collaboration: 43.4 minutes; *P*<.001). Analysis of the different surgical phases showed that the sellar phase had the lowest frequency (nasal phase: 14.9, sphenoidal phase: 15.9, and sellar phase: 4.9 interruptions/10 minutes; *P*<.001) and duration (nasal phase: 67.4, sphenoidal phase: 77.9, and sellar phase: 31.1 seconds/10 minutes; *P*<.001) of surgical interruptions. A comparison of the early and late EEA videos showed that increased surgical experience was associated with a decreased number (early: 4.9 and late: 2.9 interruptions/10 minutes; *P*=.03) and duration (early: 41.1 and late: 19.8 seconds/10 minutes; *P*=.02) of surgical interruptions during the sellar phase.

**Conclusions:**

CV-based analysis had a 93% to 98% accuracy in detecting the number, frequency, and duration of surgical interruptions occurring during EEA. Moreover, CV-based analysis reduced the time required to analyze the surgical fluency in EEA videos compared to manual review. The application of CV can facilitate the training of surgeons to overcome the learning curve of endoscopic skull base surgery.

**Trial Registration:**

ClinicalTrials.gov NCT06156020; https://clinicaltrials.gov/study/NCT06156020

## Introduction

The endonasal endoscopic approach (EEA) is an effective technique for pituitary adenoma (PA) resection [1-3]. The introduction of an operative endoscope into the nasal cavity greatly improves the intraoperative visualization of critical neurovascular structures and facilitates the resection of tumors with suprasellar and cavernous sinus components [1,4]. Compared with microscopic transsphenoidal resections, EEA has been shown to have a higher percentage of complete resection and a lower incidence of neurovascular complications when performed by experienced surgeons [4-6]. Nevertheless, EEA has a long learning curve compared with microscopic approaches. A previous study reported that a 15- to 50-case learning curve is required to improve EEA outcomes [7,8].

To shorten the learning curve for endoscopic surgeries, artificial intelligence (AI) and computer vision (CV) analyses of operative videos provide novel and effective tools to reduce the time required for manual review [9]. Xu et al [10] reported the use of CV in the analysis of thoracoscopic pulmonary lobectomies and demonstrated that bleeding could be detected using CV and was associated with postoperative outcomes. Ryu et al [11] described the use of a CV-based tracking of surgical instruments in robot-assisted laparoscopic surgery to prevent instrument collisions. The use of CV analyses in endoscopic neurosurgery has been reported only recently. Khan et al [12] developed a CV-based workflow analysis to distinguish the nasal, sphenoidal, and sellar phases during EEA and achieved accurate automated recognition of the surgical phases and steps.

Although the system developed by Khan et al [12] could evaluate the time spent in each phase of EEA surgery, however, technical fluency and operative efficiency were not evaluated, which could provide invaluable information for surgeons to refine their operative techniques and improve the learning curve [13,14]. Currently, manual review of EEA operative videos remains time-consuming and ineffective [14]; therefore, an automated CV-based system to analyze the technical fluency and operative efficiency of EEA videos will facilitate the training of surgeons to overcome the learning curve of endoscopic skull base surgery.

To address this knowledge gap, this study aimed to develop a CV-based system for analyzing surgical fluency in EEA videos by detecting the number, frequency, and duration of surgical interruptions that occur during EEA surgeries. Since removal and reintroduction of the endoscope into the nasal cavity require a transition of the surgeons’ focus from the video screen to the operative field and frequent redirection of the surgeons’ attention was associated with deleterious effects on surgeons’ performance [15,16], we aimed to detect the events of removal and reintroduction of the endoscope during EEA to represent surgical interruptions. The time spent on CV-based and manual reviews of the surgical video was compared. Additionally, the learning curve of the EEA and the impact of tubular retractor use were investigated.

## Methods

### Ethical Considerations

This study was approved by the institutional review board of the National Cheng-Kung University Hospital (approval B-ER-112-452). The requirement for informed consent was waived, as approved by the institutional review board. All patient data, including the operative video, were deidentified. Compensation was not provided to included patients, as approved by the institutional review board. This study was reported in line with the STROCSS (Strengthening the Reporting of Cohort Studies in Surgery) criteria [17] and registered on ClinicalTrials.gov (NCT06156020).

### Patients

Patients who underwent EEA for PAs between January 2020 and March 2024 with fully recorded anonymous operative videos were retrospectively reviewed. We excluded patients whose operative videos were incompletely recorded and those with poor-quality operative videos. Patients were excluded if their facial identity could be recognized in the video. All patients underwent standard 2-surgeon EEA techniques performed by an ear, nose, and throat rhinology specialist and a neurosurgeon. All surgeries were performed endoscopically.

Videos from January 2020 to August 2023 were randomly assigned into training and test 1 data sets. Videos from September 2023 to February 2024 were assigned into test 2 data set, which included EEA videos recorded using a new set of operative endoscopes and surgical instruments or performed by another team of surgeons.

### Surgical Fluency and Surgical Interruptions

The definitions of surgical interruptions were reviewed and varied according to the types of different surgeries [18]. In this study, surgical interruption was defined on the basis of all of the following criteria: (1) blood contamination of endoscopic vision, (2) removal of the endoscope from the nasal cavity, and (3) cleaning of the endoscope. These criteria indicate cessation of operative maneuvers and were used for both the manual review and development of the CV-based algorithm. The number and duration of the surgical interruptions were also recorded. The frequency and total duration of the surgical interruptions were calculated every 10 minutes.

### CV-Based Algorithm

**Figure 1:**
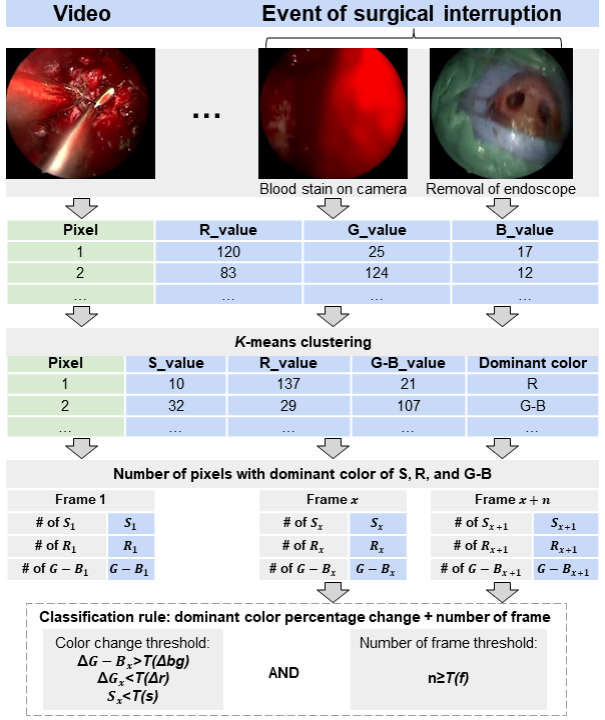
Graphical illustration of the present computer vision–based algorithm to detect a surgical interruption in endonasal endoscopic approach resection of pituitary adenomas. #: number; G–Bx: number of pixels with green-blue dominant color; RGB: red, green, and blue; Rx: number of pixels with red dominant color; SRG-B: silver, red, green and blue; Sx: number of pixels with silver dominant color.

Our algorithm aimed to detect surgical interruptions that necessitated the removal of the endoscope from the nasal cavity for cleaning (Figure 1). All surgical videos were converted to 19201080 pixels. When given a surgical video with red, green, and blue channels, the 8-bit per channel red, green, and blue values of all the pixels in each frame could be obtained using OpenCV software and expressed as a 1920×1080×3 matrix. Under normal illumination, the color signatures of the nasal and sellar mucosa and structures were red-pinkish, the surgical drape was blue-greenish, and the surgical instruments and gauze were silverish-whitish. Using this property, nasal mucosa, surgical drapes, and instruments were classified using *K*-means clustering, a variance-based clustering technique that can be used to classify different objects.

Then, the image color space was converted to the SRB-G space, where S stands for silver and R and B-G stand for the 2-color channels representing the nasal and sellar mucosa and surgical drapes, respectively. The dominant color in terms of SRB-G in each pixel could then be determined, and the number of pixels with the dominant colors S, R, and B-G could be expressed as S, R, and B-G, respectively. The alterations in the dominant color in each frame were compared to those in the previous frames and could further be expressed as ΔS, ΔR, and ΔB-G, respectively. An algorithm was developed to claim surgical interruption events according to the thresholds of S, R, and B-G and ΔS, ΔR, and ΔB-G as follows:

Δ*B–G>T*(Δ*bg*) AND Δ*R<T*(Δ*r*) AND *S<T*(*s*)

Where *T*(Δ*bg*) and *T*(Δ*r*) are the percentage thresholds of the alterations in the B-G and R values, respectively, indicating that a sufficient increase in the B-G percentage and a simultaneous decrease in the R percentage were required to claim a surgical interruption. Furthermore, *T*(*s*) was the threshold of the S percentage, which was used to exclude the impact of moving surgical instruments on alterations in R and B-G values.

Because the event of surgical interruption did not occur in a single frame but rather in consecutive frames, the S, R, and B-G values were compared to the numbers of previous frames, which we further improved the foregoing method by adding a threshold in the number of consecutive frames *T*(*f*) compared. An event of surgical interruption should last longer than this threshold; therefore, if an identified event lasted less than *T*(*f*) frames, it would not be claimed as a surgical interruption event. The thresholds *T*(Δ*bg*), *T*(Δ*r*), *T*(*s*), and *T*(Δ*f*) were determined using the training data set. We found that settings *T*(Δ*bg*) of 35%, *T*(Δ*r*) of 22%, *T*(*s*) of 32%, and *T*(*f*) of 30% optimized the performance of the foregoing detection rules on the training data set with an overall accuracy of 93.6% (sensitivity=94.7%, specificity=93.4%).

### Statistical Analysis

Parametric variables were compared using the 2-tailed Student *t* test. Nonparametric variables were compared using the Mann-Whitney *U* test. Multiple comparisons of parametric variables were performed using ANOVA. Multiple comparisons of paired variables were performed using repeated measures ANOVA with pairwise post hoc comparisons. Statistical tests were conducted using MedCalc (version 19.7.2; MedCalc Software, Ltd).

## Results

### Operative Videos Included for Analysis

In total, 46 EEA operative videos were analyzed, with 10, 26, and 10 videos used as the training, test 1, and test 2 data sets, respectively. The overall accuracy in the training data set was 93.6% (sensitivity=94.7%, specificity=93.4%). The overall accuracy in test 1 data set was 95.1% (sensitivity=94.5%, specificity=95.3%). The overall accuracy in test 2 data set was 93.3% (sensitivity=93.1%, specificity=93.4%).

**Figure 2:**
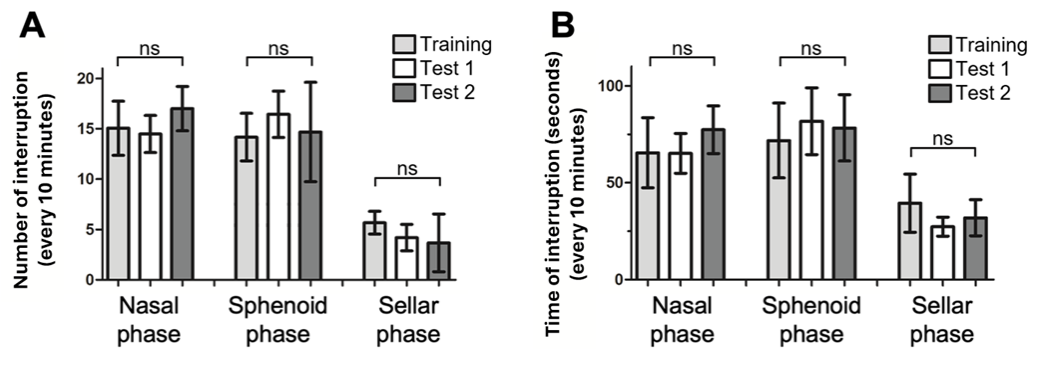
Comparison between the computer vision–based detection results between the training and test data sets. (A) The average number and (B) average total duration of surgical interruptions normalized to every 10 minutes in the nasal, sphenoidal, and sellar phases of the training and test video data sets. ns: not significant.

The number of surgical interruptions every 10 minutes was similar between the 3 data sets in all surgical phases (ANOVA, nasal phase: training=15.1, test 1=14.5, and test 2=17.0 interruptions/10 minutes; *df*=2; *P*=.65; sphenoidal phase: training=12.4, test 1=14.7, and test 2=12.9 interruptions/10 minutes; *df*=2; *P*=.37; and sellar phase: training=5.6, test 1=4.2, and test 2=3.7 interruptions/10 minutes; *df*=2; *P*=.16; Figure 2A). The duration of surgical interruption was also similar between the data sets (ANOVA, nasal phase: training=65.4, test 1=65.1, and test 2=77.6 seconds/10 minutes; *df*=2; *P*=.70; sphenoidal phase: training=71.8, test 1=81.7, and test 2=78.3 seconds/10 minutes; *df*=2; *P*=.75; and sellar phase: training=39.4, test 1=27.3, and test 2=31.9 seconds/10 minutes; *df*=2; *P*=.15; Figure 2B).

### Time Used in CV-Based and Manual Analyses

**Figure 3:**
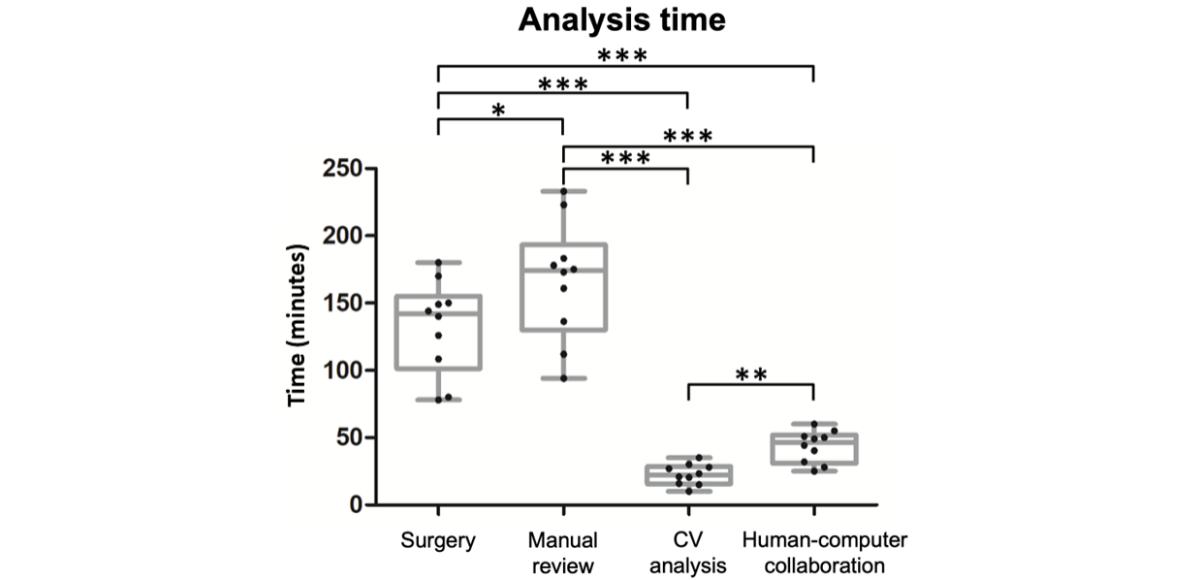
The average length of endonasal endoscopic approach videos and the time required for manual, CV-based, and human-computer collaborative video analyses. CV: computer vision. *P<.05, **P<.01, and ***P<.001.

The time required for manual, CV-based, and human-computer collaborative analyses of EEA operative videos is presented in Figure 3 (repeated measures ANOVA; *df*=27; *P*<.001). The average operative video length was 132.5 (SD 34.6) minutes, and the average time spent for manual video review was 166.8 (SD 43.8) minutes (paired post hoc comparison, surgery: 132.5 and manual review: 166.8 minutes; *P*=.01). The average time required for CV-based automated analysis was 22.6 (SD 7.6) minutes, which was significantly less than the video length (paired post hoc comparison, CV analysis: 22.6 and surgery: 132.5 minutes; *P*<.001) and the time used in the manual analysis (paired post hoc comparison, CV analysis: 22.6 and manual review: 166.8 minutes; *P*<.001).

To improve the accuracy of the current CV-based operative video analysis, we used a human-computer collaborative strategy, in which the videos were first analyzed using an automated system, and the identified events of surgical interruptions were subsequently evaluated manually to determine their correctness. In this strategy, the average time spent for analysis was 43.4 (SD 11.8) minutes, which was significantly less than the video length (paired post hoc comparison, human-CV collaboration: 43.4 and surgery: 132.5 minutes; *P*<.001) and the time used in manual analysis (paired post hoc comparison, human-CV collaboration: 43.4 and manual review: 166.8 minutes; *P*<.001) but longer than the time for the CV-based automated analysis (paired post hoc comparison, human-CV collaboration: 43.4 and CV analysis: 22.6 minutes; *P*=.002).

### The Learning Curve in the Different Surgical Phases of EEA

**Figure 4:**
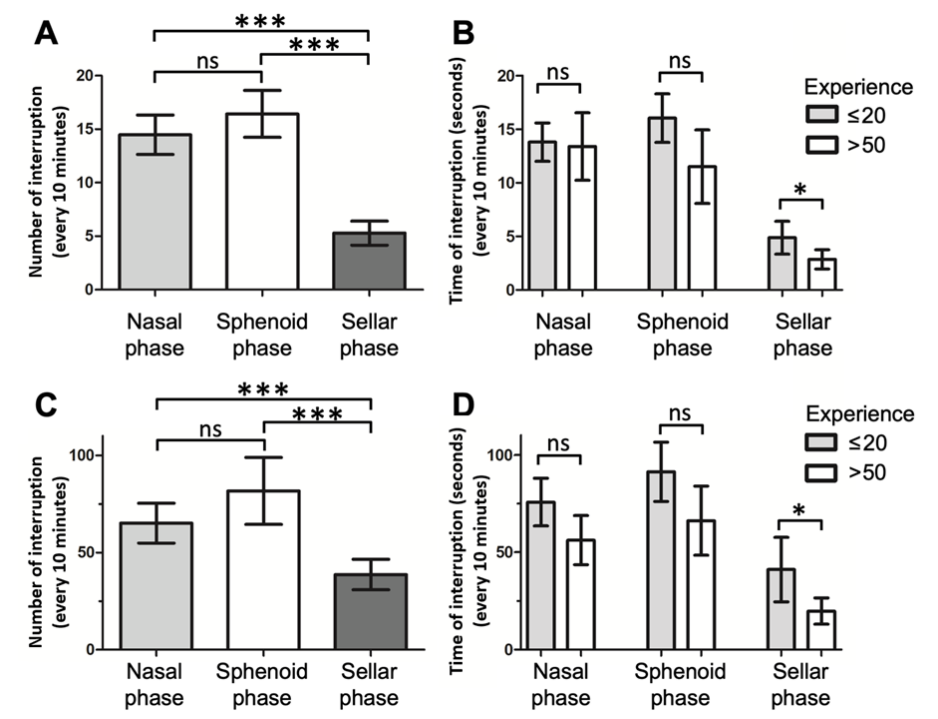
Surgical interruptions during different phases of EEA and the effect of surgeon experiences. (A) The average number and (C) the average total duration of surgical interruptions normalized to every 10 minutes in the nasal, sphenoidal, and sellar phases of EEA operative videos. Comparisons of (B) the average number and (D) the average total duration of surgical interruptions in EEA operative videos of surgeons with ≤20 procedural experiences and >50 procedural experiences. EEA: endonasal endoscopic approach.

We investigated the difference in frequency (ANOVA, nasal phase: 14.9, sphenoidal phase: 15.9, and sellar phase: 4.9 interruptions/10 minutes; *df*=2; *P*<.001) and duration (ANOVA, nasal phase: 67.4, sphenoidal phase: 77.9, and sellar phase: 31.1 seconds/10 minutes; *df*=2; *P*<.001) of surgical interruptions that occurred during the different phases of EEA (Figure 4A and C). The sellar phase had the least number and duration of surgical interruptions every 10 minutes compared with the nasal (paired post hoc comparison, sellar phase: 4.9 and nasal phase: 14.9 interruptions/10 minutes; *P*<.001) and sphenoidal phases (paired post hoc comparison, sellar phase: 4.9 and sphenoidal phase: 15.9 interruptions/10 minutes; *P*<.001). Although the sphenoidal phase had the highest number and longest duration of surgical interruptions, the differences were not significant compared with those in the nasal phase (number of interruptions: paired post hoc comparison, sphenoidal phase: 15.9 and nasal phase: 14.9 interruptions/10 minutes; *P*=.16; duration of interruptions: paired post hoc comparison, sphenoidal phase: 77.9 and nasal phase: 67.4 seconds/10 minutes; *P=*.10).

**Figure 5:**
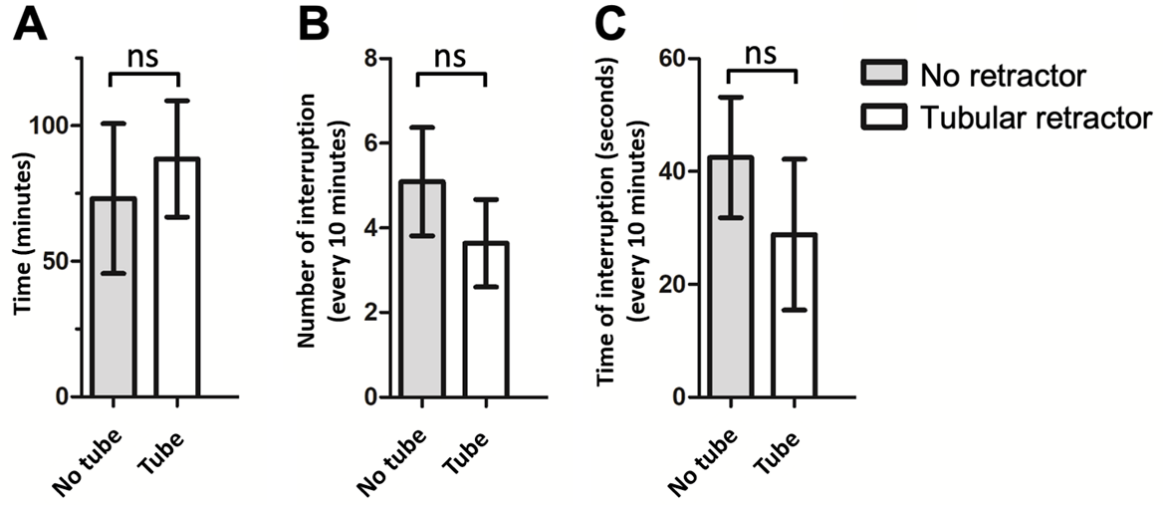
The effect of a tubular retractor in the sellar phase of endonasal endoscopic approach (EEA) operative videos. Quantitative comparisons of the (A) operative time, (B) average number, and (C) average total duration of surgical interruptions in the sellar phase of EEA operative videos with or without the use of a tubular retractor.

To demonstrate the learning curve of EEA, the analysis results of early (≤20 procedural experiences, n=11) and late operative videos (>50 procedural experiences, n=8) were compared (Figure 4B and D). The results demonstrated a decreased number (2-tailed Student *t* test, early: 4.9 and late: 2.9 interruptions/10 minutes; *P*=.03) and duration (2-tailed Student *t* test, early: 41.1 and late: 19.8 seconds/10 minutes; *P*=.02) of surgical interruptions in the sellar phase in late videos compared with early videos. Additionally, there were mild trends of fewer numbers (2-tailed Student *t* test, early: 16.0 and late: 11.6 interruptions/10 minutes; *P*=.09) and shorter durations (2-tailed Student *t* test, early: 91.2 and late: 69.0 seconds/10 minutes; *P*=.08) of surgical interruptions in the sphenoidal phase of late videos compared with early videos, but the difference was not statistically significant. The use of a tubular retractor in the sellar phase did not affect the operative time (2-tailed Student *t* test, no tube: 73.1 and tube: 87.7 minutes; *P*=.31), number (2-tailed Student *t* test, no tube: 5.1 and tube: 3.7 interruptions/10 minutes; *P*=.10), or duration (2-tailed Student *t* test, no tube: 42.4 and tube: 28.8 seconds/10 minutes; *P*=.11) of surgical interruptions (Figure 5).

## Discussion

### Principal Findings

The application of CV can improve the review process of operative videos, thereby facilitating the training of surgeons to overcome the learning curve of endoscopic skull base surgery. In this study, we developed a CV-based video analysis system to detect surgical interruptions and analyze surgical fluency in the EEA with an overall accuracy of 93.6%. The CV-based analysis greatly reduced the time required for operative video reviews. The learning curves of the different phases of EEA surgery were demonstrated in the CV-based analysis.

The algorithm used here was designed to detect events of surgical interruption. Removal and reintroduction of the operative endoscope into the nasal cavity require time and transition of the surgeons’ focus from the video screen to the operative field [16]. Frequent redirection of the surgeons’ attention during surgery was associated with increased surgeon load on working memory, and intraoperative distraction was associated with a deleterious effect on the surgeons’ performance [15,19,20]. Moreover, re-entering the endonasal corridor implies the need for reidentification of critical neurovascular structures, such as the optic nerves and internal carotid artery; thus, frequent endoscope removal and re-entry into the endonasal corridor might increase the risk of misidentification and neurovascular injury [16]. Therefore, the frequency and duration of surgical interruptions are critical intraoperative factors that can affect surgical safety and are used as indicators of surgical fluency in this study. Our results showed that the total amount of time spent in surgical interruption could approach 90-120 seconds every 10 minutes, which was equal to 15% to 20% of the time. A reduction in surgical interruption could potentially reduce both the operative time and the risk of surgical complications [18,21]. Although other parameters, such as instrument collision, were reported as indicators of surgical fluency in thoracoscopic and laparoscopic surgeries [22,23], they were not suitable for the EEA because instrument collision might occur frequently considering the narrow endonasal corridor of the approach.

### Comparison to Prior Work

The method used in the present algorithm to detect surgical interruptions was primarily based on the identification of the dominant colors in each pixel [24]. Similar approaches were used by Xu et al [10], who designed a CV-based algorithm to detect bloodstains in thoracoscopic surgeries by identifying red pixels. The use of color-based recognition is a reasonable and effective approach, especially for detecting a surgical interruption in the EEA, because the removal of the endoscope from the nasal cavity creates a considerable color contrast between the reddish-pinkish nasal mucosa and greenish-bluish surgical drapes, which facilitates CV recognition of alterations in the dominant color [25,26]. Moreover, considering the aforementioned color spectra analyzed during the EEA, we transformed the original R-G-B color information into the SRG-B space. Instead of using only 2-color channels, we added an S channel to minimize the interference generated by the silverish surgical instrument and whitish gauze in the video [11]. Combining these approaches together, the present algorithm was able to achieve an overall accuracy of 93.6%. Furthermore, the accuracy of the CV-based method can be further improved using a human-computer collaborative strategy [27,28]. In our approach, videos are first analyzed using an automated system, and the identified surgical interruption events are subsequently evaluated manually to determine their correctness. Although the unidentified (false-negative) surgical interruptions could remain undetected, the false-positive surgical interruptions could be eliminated, and the overall accuracy was increased to 98.5%.

### Strengths and Limitations

The main goal of the present CV-based EEA video analysis system is to reduce the time spent reviewing operative videos. Previous applications of CV-based videos in PA surgeries were mainly aimed at recognizing the different phases and determining the surgical workflow [12,29]. Lalys et al [29] used feature extraction and principal component analysis to classify the microscopic resection of PA into 6 phases, with the best correct classification rate of 82%. Similarly, Khan et al [12] developed an automatic CV-based workflow analysis of endoscopic resection of PAs and successfully classified surgical workflow into 3 phases and 7 steps, with accuracies of 90% and 75%, respectively. However, in these studies, the authors did not report the time required for automated analysis or the time saved compared with manual analysis. Therefore, the efficiency of the system remains to be evaluated. Compared with the classification of different surgical phases, the identification of surgical interruption events throughout surgery is potentially a much more time-consuming task [30]. In manual analysis, a review of the entire length of the operative video is required to recognize all the interrupted events. Moreover, additional time was spent recording the events. Therefore, it is reasonable that our results showed that the time required for manual review was significantly longer than the original length of the video [30]. Therefore, the recognition of surgical interruption is a time-consuming task and is well suited for CV-based analysis to reduce the analysis time. Our results showed that the use of CV-based analysis significantly reduced the time required for analysis by approximately 86%. Although the use of the human-computer collaborative strategy slightly increased the time, there was still a 74% reduction in the time spent for operative video analysis. Hence, these results demonstrate that the primary goal of the CV-based analysis was successfully achieved. The application of the present CV-based system will facilitate the training of surgeons to overcome the learning curve of endoscopic skull base surgery more efficiently.

Moreover, the present CV-based analysis could be further used to characterize the learning curve of the EEA and compare different surgical techniques. A previous study reported that a 15- to 50-case learning curve is required in EEA surgeries [7,8,31]. Previous studies by Lofrese et al [8] and Qureshi et al [31] reported a decreased operative duration with increasing experience. Leach et al [7] reported improvements in the duration of surgery and visual outcomes after approximately 50 procedures. However, conflicting results have been reported regarding operative outcomes, with some studies reporting no correlation between surgeon experience and complication rates [8]. Furthermore, confounding factors such as tumor size could affect the surgical length and patient outcome [32-34]. Therefore, the analysis of operative fluency and the calculation of surgical interruption normalized by unit time provided a more objective and quantitative approach to characterize surgical learning curves [18]. Here, we showed that increased experience was associated with decreased surgical interruptions in the sellar phase of the EEA for PA resection. Since the nasal phase and the initial sphenoidotomy were performed by an expert ear, nose, and throat surgeon who specializes in functional endoscopic sinus surgery, it is reasonable that the experience in the 2-surgeon 3-hand approach has less of an effect on surgical fluency in the nasal and sphenoidal phase [35]. In contrast, our results clearly demonstrated the effect of the learning curve on the duration of surgical interruptions in the sellar phase. Additionally, although the use of tubular retractors did not result in a significant reduction in surgical interruptions, these results demonstrate that CV-based analysis of EEA videos can be applied to compare the impact of different surgical techniques or the use of adjunctive surgical instruments on surgical fluency.

This study had several limitations. First, we included operative videos from a single institution. Nonetheless, we analyzed 2 separate test data sets, which comprised EEA videos recorded using different sets of operative endoscopes and surgical instruments or performed by other teams of surgeons to better demonstrate the generalizability of the present CV algorithm. Still, further investigation with EEA videos from other institutions was required to externally validate the present CV-based analysis method. However, since the present CV-based analysis relied mainly on the identification of the dominant color, the effect of interinstitutional differences is potentially minimized, given the similarity in the colors of the nasal mucosa and surgical drapes. Second, additional intraoperative factors could have also influenced the accuracy of our CV analysis as well as the frequency of surgical interruptions. These factors included hypertrophic nasal mucosa, a deviated nasal septum, intraoperative hemorrhage, and the use of novel surgical instruments, which could result in misidentification and bias in analyzing the EEA learning curve [36-38]. Nonetheless, this CV-based analysis could be used to investigate the effects of these intraoperative factors. Further, we showed that the application of human-computer collaborative strategy could increase the overall accuracy to overcome this limitation. Third, the CV method used in this study was an image processing algorithm based on OpenCV color detection rather than a trained, machine learning AI model. The use of machine learning AI model in surgical analysis could potentially extract more information from the operative videos. However, conventional image processing offered several advantages such as faster inference on computer central processing unit and elimination of the need for training on expensive graphic processing units. Moreover, we were able to achieve a 93.3% to 95.1% overall accuracy in this analysis. Finally, whether the frequency and duration of surgical interruptions are associated with patient outcomes remains unclear. Future studies with comprehensive analyses are warranted to clarify the effects of surgical interruptions and other patient and intraoperative factors on patient outcomes.

### Conclusions

This study developed a CV-based system to analyze surgical fluency in EEA videos by detecting the number, frequency, and duration of surgical interruptions that occur during EEA surgeries. The CV-based video analysis system had an overall accuracy of 93.6% and significantly reduced the time required for operative video review. The application of CV can facilitate the training of surgeons to overcome the learning curve of endoscopic skull base surgery.

## Abbreviations

AI: artificial intelligence
CV: computer vision
EEA: endonasal endoscopic approach
PA: pituitary adenoma
STROCSS: Strengthening the Reporting of Cohort Studies in Surgery

## Data Availability

The data sets generated and analyzed during this study are not publicly available due to institutional board regulations but are available from the corresponding author on reasonable request.
